# Label-free discrimination of tumorigenesis stages using in vitro prostate cancer bone metastasis model by Raman imaging

**DOI:** 10.1038/s41598-022-11800-w

**Published:** 2022-05-16

**Authors:** Sumanta Kar, Sharad V. Jaswandkar, Kalpana S. Katti, Jeon Woong Kang, Peter T. C. So, Ramasamy Paulmurugan, Dorian Liepmann, Renugopalakrishnan Venkatesan, Dinesh R. Katti

**Affiliations:** 1 Department of Civil, Construction and Environmental Engineering, Center for Engineered Cancer Testbeds, Materials and Nanotechnology Program, North Dakota State University, Fargo, ND 58108 USA; 2 Laser Biomedical Research Center, G. R. Harrison Spectroscopy Laboratory, Massachusetts Institute of Technology, MB 02139 Cambridge, USA; 3 Cellular Pathway Imaging Laboratory (CPIL), Department of Radiology, Stanford University School of Medicine, 3155 Porter Drive, Suite 2236, Palo Alto, CA 94304 USA; 4 Department of Bioengineering, University of California, Berkeley, CA USA; 5 Boston Children’s Hospital, Harvard Medical School, Boston, MA 02115 USA; 6 Department of Chemistry and Chemical Biology, Northeastern University, Boston, MA 02115 USA

**Keywords:** Cancer imaging, Cancer models, Metastasis, Tumour biomarkers

## Abstract

Metastatic prostate cancer colonizes the bone to pave the way for bone metastasis, leading to skeletal complications associated with poor prognosis and morbidity. This study demonstrates the feasibility of Raman imaging to differentiate between cancer cells at different stages of tumorigenesis using a nanoclay-based three-dimensional (3D) bone mimetic in vitro model that mimics prostate cancer bone metastasis. A comprehensive study comparing the classification of as received prostate cancer cells in a two-dimensional (2D) model and cancer cells in a 3D bone mimetic environment was performed over various time intervals using principal component analysis (PCA). Our results showed distinctive spectral differences in Raman imaging between prostate cancer cells and the cells cultured in 3D bone mimetic scaffolds, particularly at 1002, 1261, 1444, and 1654 cm^−1^, which primarily contain proteins and lipids signals. Raman maps capture sub-cellular responses with the progression of tumor cells into metastasis. Raman feature extraction via cluster analysis allows for the identification of specific cellular constituents in the images. For the first time, this work demonstrates a promising potential of Raman imaging, PCA, and cluster analysis to discriminate between cancer cells at different stages of metastatic tumorigenesis.

## Introduction

Prostate cancer is the most frequently diagnosed cancer amongst men. According to recent statistics, the 5-year survival rate of patients with primary prostate cancer is 100% when diagnosed early, while it declines to 30% in the case of metastases^1^. Metastatic prostate cancer colonizes in the bone marrow to pave the way for bone metastasis, leading to cytoskeletal complications and poor prognosis and morbidity. There has been an increasing demand for translational models that recapitulate bone metastasis of prostate cancer to understand the mechanisms that underlie bone metastasis, mainly due to the lack of availability of human prostate cancer metastasized bone samples and in vivo mouse models for spontaneous prostate cancer bone metastasis. Moreover, the complexity of this disease itself poses a challenge for the diagnosis and assessment of populations with therapeutic resistance. The basic histopathological examination is the current gold standard for determining the prognosis of prostate cancer in patients. Immunohistopathology has been used extensively for the analysis of biopsy samples in the last decade. Nevertheless, immunohistochemistry has limitations due to the difficulty of analyzing large volumes of tissue sections by staining and the inability to detect multiple signals simultaneously. Also, the reproducibility and robustness of genomic data remain a concern due to the heterogeneity of tumors. It is, therefore, necessary to develop robust diagnostic and classification tools that have reproducibility and translational application with clinical samples.

Raman spectroscopy is an inelastic scattering phenomenon used to probe molecular vibrations, thus providing a molecular fingerprint that is especially suited for biological macromolecules. This technique is based on a change in polarizability of a molecule on the absorption of photons. In contrast, complementary infrared (IR) spectroscopy depends on a change in the dipole moment. Raman spectroscopy is an information-rich spectroscopic method, like FT-IR, capable of detecting specific groups and the environment-sensitive cell surface composition of biological macromolecules consisting of carbohydrates, proteins, and lipids, among others^2–4^. A recent study demonstrated the feasibility of the complementary vibrational spectroscopy technique for accurate and precise chemical analysis of organic liquids by combining IR and Raman spectroscopy^5^. Raman spectroscopy has been widely used to differentiate between normal, nonmalignant, and malignant cell lines, from breast^6,7^, colorectal^8^, gastrointestinal^9^, lung^10^, and prostate cancer cells/tissues^11–13^. Recent studies demonstrate the use of Raman spectroscopy to distinguish between the osteoblastic and osteolytic bone metastases^14^. A few studies have addressed stage-based classifications^15,16^. A recent study demonstrates the use of Raman mapping to evaluate sub-cellular responses in prostate cancer cells (PC-3) to X-ray exposure while also elucidating the advantage of Raman mapping techniques over single-point measurement^17^. Also, recent studies demonstrate the use of Raman spectroscopy and artificial intelligence to evaluate the probability of breast cancer in a clinical environment^18^. A recent study describes the use of optical diffraction tomographic imaging and Raman spectroscopy to combine morphological and molecular data for phenotyping breast cancer cells^19^. Furthermore, most previous studies used Raman spectroscopy in prostate cancer research based on two-dimensional (2D) culture models^15,20^. It has been reported that in the 2D system, cells lose their in vivo characteristics, resulting in poor cell–cell and cell–matrix interactions^21^. In contrast, three-dimensional (3D) in vitro models recapitulate a physiological environment that mimics in vivo conditions, leading to improved predictions^22^. The primary goal of this study is to evaluate Raman spectroscopy as a potential tool for monitoring cancer progression at bone metastasis using a nanoclay-based 3D bone mimetic in vitro model of prostate cancer bone metastasis. For this purpose, we developed a 3D bone mimetic in vitro model^23^ using nanoclay-based scaffolds^24^ along with osteogenically differentiated human mesenchymal stem cells (MSCs) and human prostate cancer cells. Our group has investigated nanoclays extensively to prepare polymer clay nanocomposites^25,26^ using both experimental and simulation-based studies, with applications towards bone tissue engineering scaffolds^27^. Our previous studies have shown osteogenic differentiation of human MSCs into osteoblasts and MSCs mediated mineralization through the biomimetic process of vesicular delivery on nanoclay based scaffolds without using osteogenic supplements^24,28^. Recently, we have reported the late stage of prostate and breast cancer pathogenesis to the bone using the sequential culture of MSCs with human prostate and breast cancer cells on nanoclay based scaffolds^29–31^. In another study, Wnt/β-catenin signaling has been shown to play a key role in osteogenesis in the 3D bone mimetic in vitro model of breast cancer bone metastasis^32^. We have also shown the influence of prostate cancer phenotype on bone mineralization at metastases^33^. In a related study from our laboratory, we have evaluated the feasibility of FT-IR spectroscopy for monitoring breast cancer progression on the 3D bone mimetic in vitro model of breast cancer bone metastasis^34^. A recent study showed how bone microenvironment secreted cytokines confer chemoresistance in breast cancer cells at bone metastases^35^. We have also described the mechanobiology of breast cancer cells^36^ and prostate cancer cells^37^ with time at the bone metastasis site using the 3D bone mimetic in vitro model.

In the present study, we describe the feasibility of Raman spectroscopy as a powerful imaging method for differentiating prostate cancer cells from prostate cancer bone metastasis tumor cells and discerning cancer progression at the bone site. We are using 2D and 3D bone mimetic cell culture models in conjunction with the application of principal component analysis (PCA) and cluster analysis. The present work addresses the: (1) potential of Raman imaging to discriminate between cancer cells at different stages of tumorigenesis; and (2) determination of key biochemical components that account for the differences observed in the cells at different stages of cancer progression as biomarkers for future prognostic application of prostate cancer bone metastasis. This is the first study that uses label-free Raman spectroscopy to differentiate tumor tissues of various stages by imaging a large tumor tissue. Previous studies^38,39^ have only used cells/tissues targeted using antibodies or ligands labelled using SERS particle for signal enhancement.

## Results and discussion

### Raman spectra of prostate cancer cells grown in 2D and 3D sequential cultures

**Figure 1:**
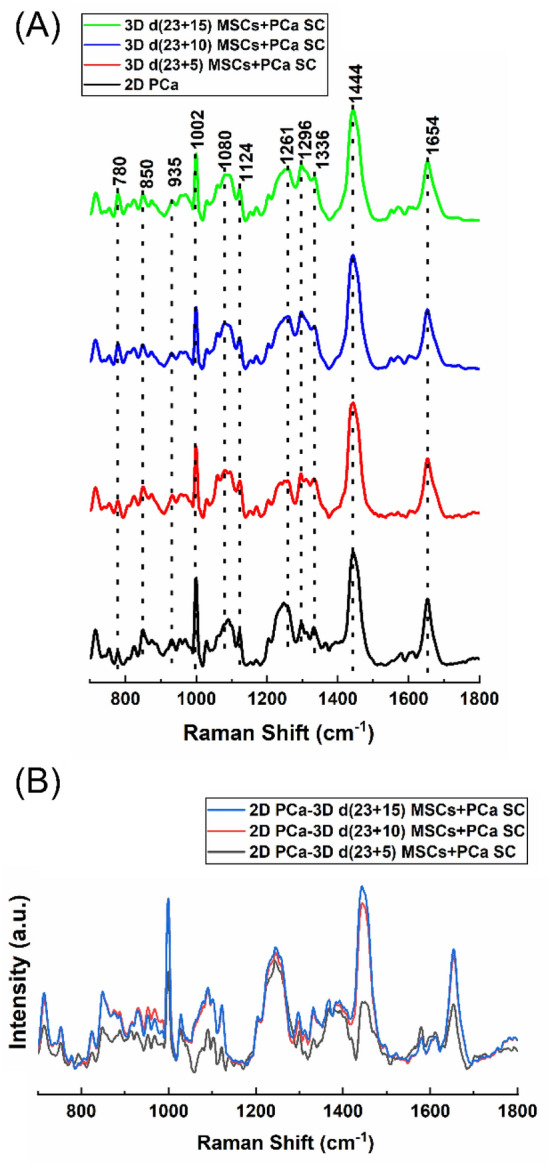
Raman spectra of prostate cancer cells. (**A**) Averages of 20 spectra from 4 samples (2D PCa and 3D MSCs + PCa SCs d(X + Y), where X = 23 days of MSCs culture and Y = 5, 10, and 15 days of cancer cell culture after 23 days of MSCs culture) are shown in bold and overlaid on representative examples of spectra for each sample. Spectra are color-grouped according to culture type and duration of culture. (**B**) The corresponding difference spectra were calculated from the averaged Raman spectra between 2D culture and three different 3D cultures.

In this manuscript, the sequential culture samples on bone mimetic scaffolds are referred to as 3D d(X + Y) MSCs + PCa SC, while 2D cancer cell culture is referred to as 2D PCa, where X = 23 days of MSCs culture and Y = 5, 10, and 15 days of cancer cell culture after 23 days of MSCs culture. The mean Raman spectra of 2D PCa, 3D d (23 + 5) MSCs + PCa SC, 3D d (23 + 10) MSCs + PCa SC, and 3D d (23 + 15) MSCs + PCa SC are shown in Fig. 1A. As seen, prominent Raman bands are observed in both 2D and 3D bone mimetic cultures of prostate cancer cells at the following peak positions with tentative biochemical assignments: 780 cm^−1^ (ring breathing of cytosine and thymine), 850 cm^−1^ (*v*(C–C) ring breathing of tyrosine and proline), 935 cm^−1^ (*v*(C–C) of α-helix conformation for proteins), 1002 cm^−1^ (*v*(C–C) ring breathing of phenylalanine), 1080 cm^−1^ (*v*(C–C) of lipids), 1124 cm^−1^ (*v*(C–O) in carbohydrates), 1261 cm^−1^ (amide III δ(N–H) of proteins), 1296 cm^−1^ (δ(CH_2_) deformations of lipids and proteins), 1336 cm^−1^ (CH_3_CH_2_ twisting of nucleic acids), 1444 cm^−1^ (δ(CH_2_) of proteins and lipids), and 1654 cm^−1^ (amide I *v*(C=O) of proteins, α- helical conformation)^20,40–45^ (Table 1). The difference in the corresponding spectra of different cultures (i.e., 2D and 3D) (Fig. 1B) reveals the Raman spectral changes, particularly in the spectral ranges of 780–850 cm^−1^, 935–1124 cm^−1^, 1261–1336 cm^−1^, 1440–1450 cm^−1^, and 1500–1800 cm^−1^ which are primarily attributed to proteins, DNA, and lipids.

**Table 1:** Band assignments for Raman spectra for prostate cancer cells^14,30–35^.

Raman shift (cm^−1^)	Band assignment
780	Ring breathing of cytosine and thymine
850	*v*(C–C) ring breathing of tyrosine and proline
935	*v*(C–C) of α-helix conformation for proteins
1002	*v*(C–C) ring breathing of phenylalanine
1080	*v*(C–C) of lipids
1124	*v*(C–O) in carbohydrates
1261	amide III δ(N–H) of proteins
1296	δ(CH_2_) deformations of lipids and proteins
1336	CH_3_CH_2_ twisting of nucleic acids
1444	δ(CH_2_) of proteins and lipids
1654	amide I *v*(C=O) of proteins, α- helical conformation

### Raman data classification, visualization, and intensity calculation

**Figure 2:**
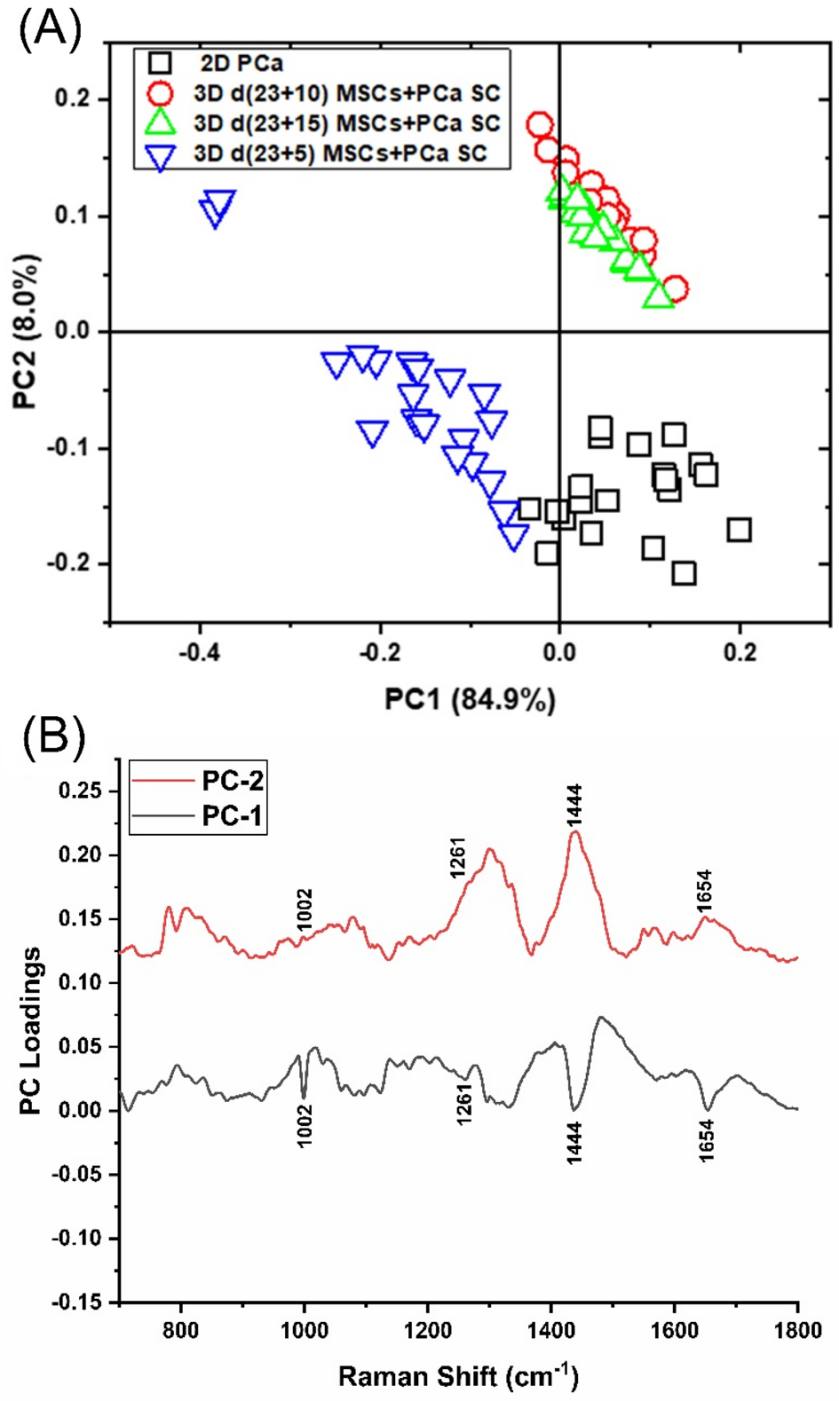
PCA analysis of Raman spectra. (**A**) PCA score plot spanned by PC1 and PC2, illustrating the intrinsic clustering of 2D PCa and 3D MSCs + PCa SCs. (**B**) The first two PCs accounting for 92.9% of the total variation in the Raman spectral dataset, revealing the significant Raman spectral features for the classification of cancer cells.

**Figure 3:**
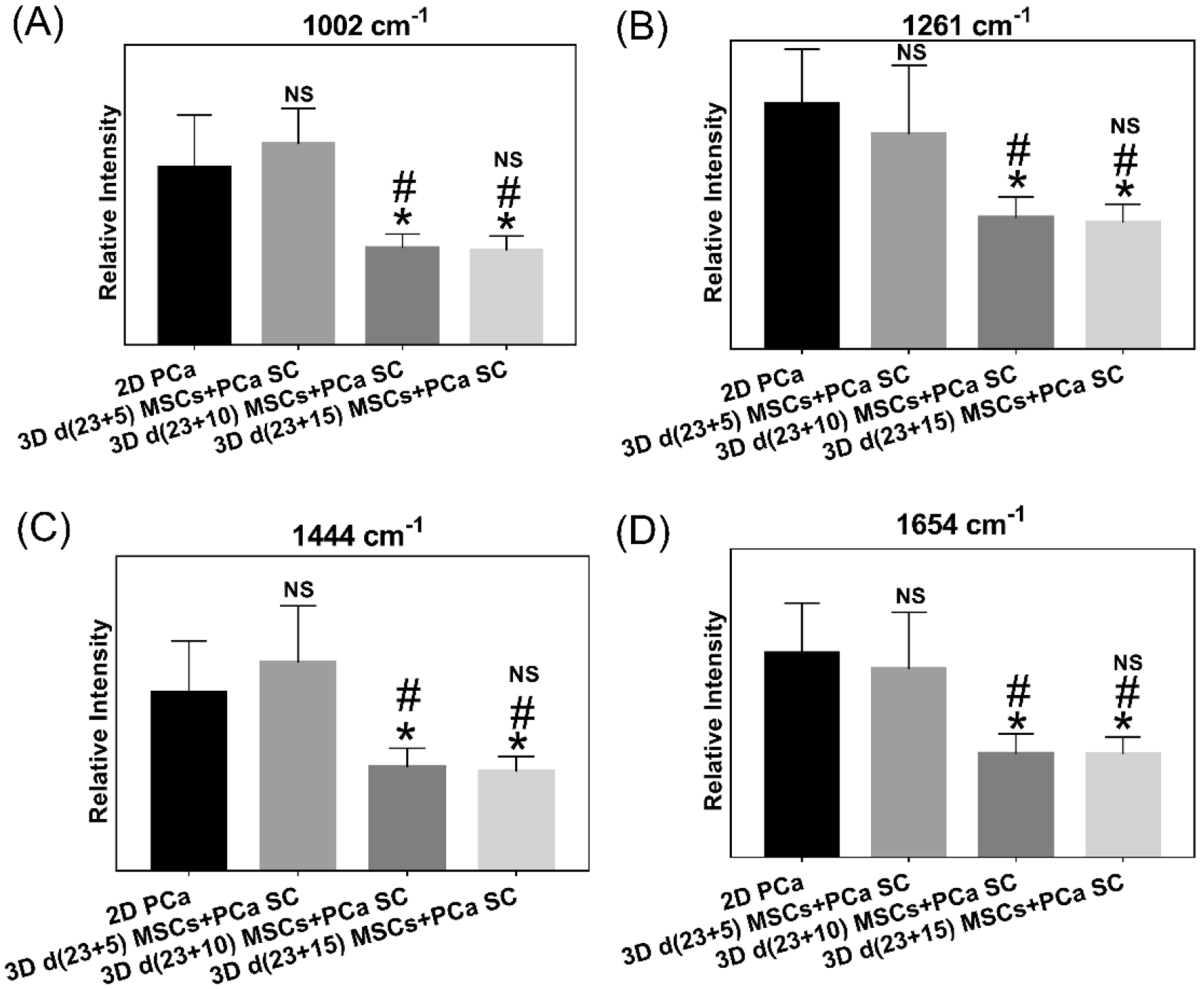
Calculation of Raman band intensity. Twenty cell spectra from each sample were selected for intensity calculation of chosen Raman bands. All spectra were normalized to DNA band at 780 cm^−1^ before analysis. Histogram displaying intensities of Raman bands (**A**) 1002 cm^−1^, (**B**) 1261 cm^−1^, (**C**) 1444 cm^−1^, and (**D**) 1654 cm^−1^ across samples. (n = 20, one-way ANOVA followed by post hoc Tukey test, *p < 0.05, **p < 0.01, and ***p < 0.001 indicate significant difference between 2D PCa and 3D MSCs + PCa SCs; ^#^*p* < 0.05, ^##^*p* < 0.01, and ^###^*p* < 0.001 indicate significant difference between day (23 + 5), and other days of 3D MSCs + PCa SCs; ^$^*p* < 0.05, ^$$^*p* < 0.01, and ^$$$^*p* < 0.001 indicate significant difference between day (23 + 10) 3D MSCs + PCa SC and day (23 + 15) 3D MSCs + PCa SC).

**Figure 4:**
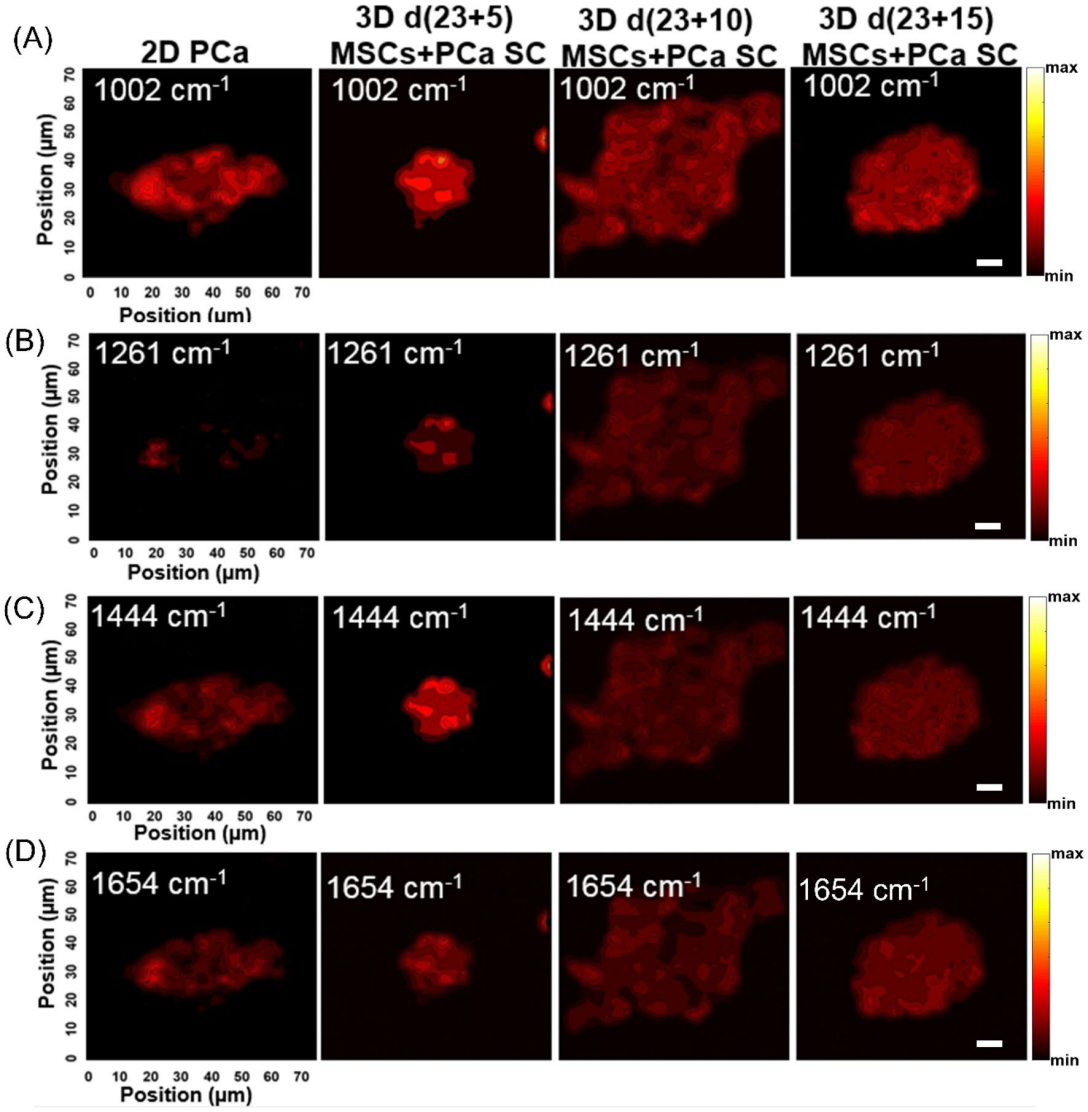
Raman maps of prostate cancer cells. Raman mapping images obtained from 2D PCa and three 3D MSCs + PCa SCs for vibrational signatures of: (**A**) 1002 cm^−1^, (**B**) 1261 cm^−1^, (**C**) 1444 cm^−1^, and (**D**) 1654 cm^−1^. Scale bars, 10 µm (**A**–**D**).

**Figure 5:**
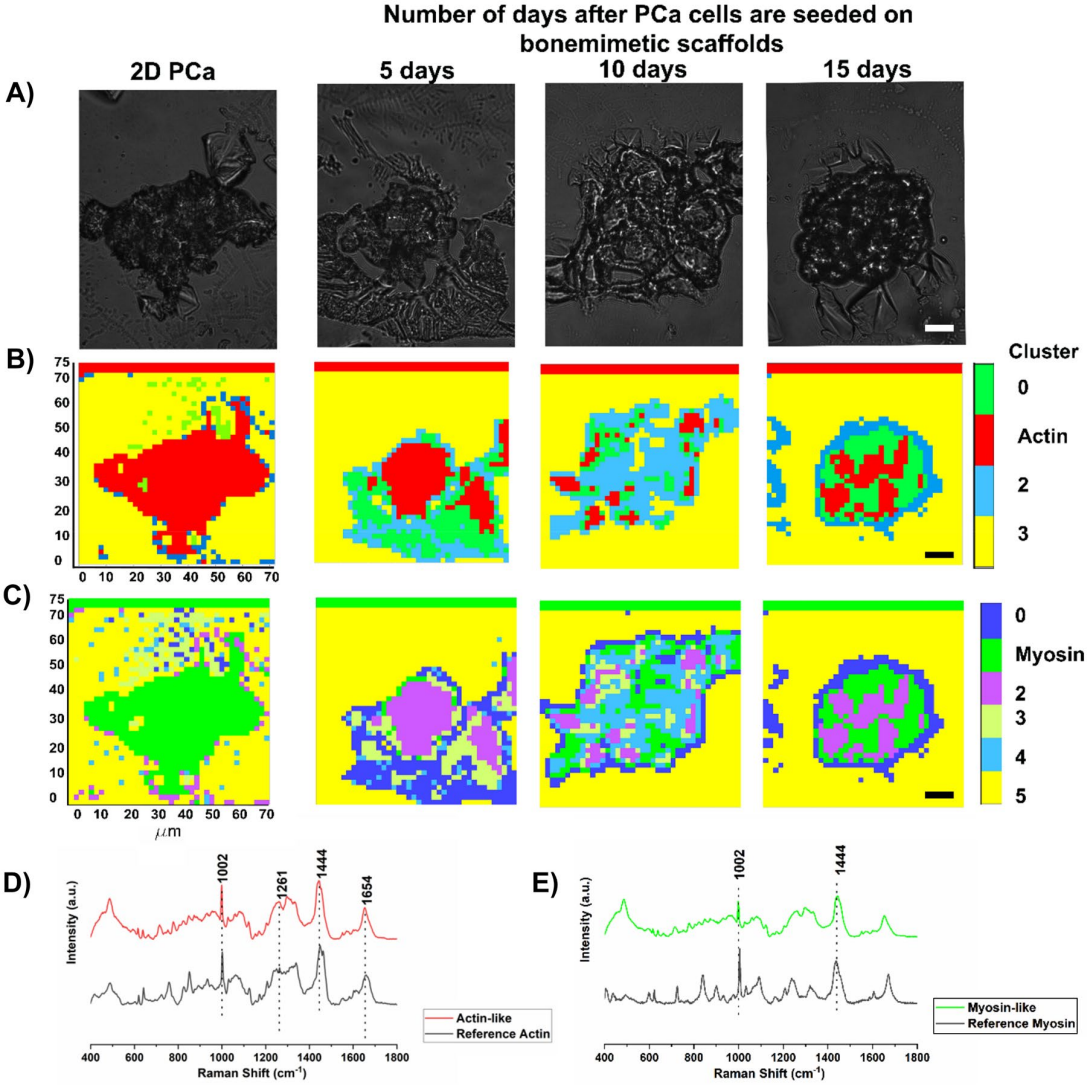
Cluster analysis of 2D PCa and PCa cells seeded on 3D bone-mimetic scaffolds for 5, 10, and 15 days. (**A**) optical images of samples used in Raman Imaging, (**B**) clusters indicating actin-rich regions colored in red, and the red stripe on the top of the image constitutes digitally introduced reference actin. (**C**) clusters indicating DNA rich regions colored in blue and the blue stripe on the top of the image constitutes digitally introduced reference DNA. Scale bars, 10 µm (**A**–**C**). (**D**) average spectra from ten randomly collected spectra from the actin-rich regions on day 15 and the reference spectra for actin, (**E**) average spectra from ten randomly collected spectra from the DNA rich regions on day 15 and the reference spectra for actin. The bone-mimetic scaffolds are created by seeding MSCs on 3D nanoclay based scaffolds and culturing for 23 days for the regeneration of bone tissue. PCa cells are seeded after the MSC culture period of 23 days is completed. The 2D PCa cells indicate PCa cells not seeded on scaffolds.

To evaluate spectral variance across the samples studied, we used principal component (PC) analysis, whose score and loading plots showed subtle differences, as shown in Fig. 2. Figure 2A displays a score plot for the first two PCs illustrating good clustering for different cultures of PCa, while Fig. 2B shows the first two PC loadings (i.e., PC1 and PC2) accounting for the largest Raman spectral variance (84.9% and 8.0%) and generally represent variations in the six sub-region bands at 1002 cm^−1^, 1261 cm^−1^, 1444 cm^−1^, and 1654 cm^−1^ in the Raman spectra. A comparison of the Raman spectra intensities ± SD at each of the four identified spectral sub-regions for all sample types studied is illustrated in the bar chart in Fig. 3. There was no appreciable change observed in intensities at all four bands (1002 cm^−1^, 1261 cm^−1^, 1444 cm^−1^, and 1654 cm^−1^) between 2D PCa and 3D d(23 + 5) MSCs + PCa SC; however, we observed a significant reduction in intensities at all four bands for 3D d(23 + 10) MSCs + PCa SC and 3D d(23 + 15) MSCs + PCa SC, as compared to both 2D PCa and 3D d(23 + 5) MSCs + PCa SC as shown in Fig. 3A–D. To visually identify the chosen Raman bands with higher specificity in the set of samples, we created surface Raman maps shown in Fig. 4, while optical micrographs for the same set of samples are shown in Fig. 5A. Only a relative determination of concentrations is possible for multi-constituent biological samples such as cells in a Raman map. Hence, we used pseudo-coloring to visually show the differences in concentration, which is proportional to the intensity of the constituent peak detected. We observed a reduction in 1002 cm^−1^ signal in 3D d(23 + 10) MSCs + PCa SC and 3D d(23 + 15) MSCs + PCa SC, as opposed to 3D d(23 + 5) MSCs + PCa SC or 2D PCa, indicating a decrease in the amount of phenylalanine relative to the total Raman-active constituents in cancer cells during cancer progression (Figs. 3A, 4A). Previous studies have reported a reduction in Raman signal at 1002 cm^−1^ in cancerous nasopharyngeal tissue compared to non-cancerous counterparts^46^. The change in the Raman band at 1002 cm^−1^ is attributed to the alteration in protein content and structural conformation. Furthermore, the Raman band at 1002 cm^−1^ has been demonstrated as a prominent and stable signal^15,39,46^. A recent study showed that blood samples of patients with advanced-stage nasopharyngeal cancer had lower phenylalanine content than that of the same diagnosed at early-stage^47^. Therefore, the band at 1002 cm^−1^ can serve as a biomarker to differentiate between stages of cancer progression at the bone site. In recent years, the cytoplasmic lipid droplets in cancer cells have received a great deal of attention owing to their ability in providing energy storage, which is supposed to be higher in rapidly growing cancer cells, that can be accessed by normal cells without the need for extra energy investment in biosynthesis^48^. These lipid droplets get absorbed, metabolized, and transformed by the cancer cells into cell membranes and other components needed for their further proliferation^49^. In line with these observations, we found a significant reduction in the intensity of the lipid-related band at 1444 cm^−1^ in 3D d(23 + 10) MSCs + PCa SC and 3D d(23 + 15) MSCs + PCa SC as opposed to 3D d(23 + 5) MSCs + PCa SC (Figs. 3B, 4C), indicating a reduction in saturated CH_2_ bonds in lipids during cancer progression^50^. Raman intensity changes at bands 1261 cm^−1^ and 1654 cm^−1^ (Figs. 3B,D, 4B,D) are associated with a change in the relative amount of proteins in the α-helical conformation. In the present study, we observed a significant reduction in intensities of the bands mentioned above in 3D d(23 + 10) MSCs + PCa SC and 3D d(23 + 15) MSCs + PCa SC as compared to 3D d(23 + 5) MSCs + PCa SC. This reduction in intensity suggests a change in protein conformation or secondary structure, i.e., α-helix to random coil conversion of proteins that play a crucial role during cancer progression^51^.

### Raman feature extraction via cluster analysis

We developed a new computational technique to rapidly identify specific constituents such as actin and myosin in cells. A strip of the pure spectra of the constituent of interest is created digitally outside the Raman image. PCA was conducted for the image, including the component strip. Next, we used the CDP Rodriguez method^52^ to cluster data into groups. If data in the specimen image is in the same cluster as the pure component stripe (actin or myosin), those regions within the map were considered similar to the component of the strip. Figure 5B shows the clusters with the actin strip at the top for the specimen from 2D, 5-day, 10-day, and 15-day tumor samples. Figure 5C shows the clusters with myosin strip at the top for the specimens from 2D, 5-day, 10-day, and 15-day tumor samples of 3D culture. Both spatial and quantitative changes to the actin cytoskeleton are observed during cancer progression and actin dynamics, leading to the reorganization of actin within the cancer cells that plays an important role in migration and the EMT/MET transitions^53^. We observed changes to the spatial distribution of both actin and myosin from 2D to the 15-day 3D tumor samples. The uniform actin and myosin distribution within cells, as observed in 2D samples, are disturbed as the tumor grows (Fig. 5B,C). We observed changes to the actin region and distribution between the 2D PCa cells and the PCa cells seeded on the bone-mimetic scaffolds (Fig. 5B). The actin regions in the cells 5-day, 10-day, and 15 days after seeding on the bone-mimetic scaffolds are smaller than the 2D PCa cells. The actin regions in the cells that were seeded on the bone-mimetic samples for 10 days and 15 days not only show the reduced size of the actin region but were also scattered and not localized. Figure 5D shows pure spectra of actin labeled as reference spectra. The average of 10 spectra obtained from the actin-rich regions in the cluster analysis is labeled as actin-like spectra. Similarly, Fig. 5E shows the myosin reference spectra and the myosin-like spectra, an average spectrum obtained from ten spectra in the myosin-rich regions in the cluster analysis. The actin-like and myosin-like spectra were obtained from the cells extracted from tumors formed 15 days after PCa cells were seeded on the bone-mimetic scaffolds. The spectra from the reference stripes of actin and myosin are not used for obtaining the actin-like and myosin-like spectra. Both the actin-like spectra and the myosin-like spectra are influenced by various other cellular constituents that would likely cause the addition of bands or degradation or amplification of intensities in some spectral regions. To validate the spectra obtained from actin-rich and myosin-rich regions in the cluster analysis, we compared them with pure actin and pure myosin reference spectra and observed actin-related bands at 1002 cm^−1^ (*v*(C–C) ring breathing of phenylalanine), 1261 cm^−1^ (amide III δ(N–H) of proteins), 1444 cm^−1^ (δ(CH_2_) of proteins), and 1654 cm^−1^ (amide I *v*(C=O) of proteins, α-helical conformation)^54^ (Fig. 5D) and myosin-related bands at 1002 cm^−1^ (*v*(C–C) ring breathing of phenylalanine), 1444 cm^−1^ (δ(CH_2_) of proteins), 1654 cm^−1^ (amide I *v*(C=O) of proteins, α-helical conformation) (shifted from 1670 cm^−1^ in reference myosin spectra) (not shown), and 1336 cm^−1^ (CH_3_CH_2_ twist) (shifted from 1320 cm^−1^ in reference myosin spectra) (not shown)^54,55^ (Fig. 5E) in actin-like and myosin-like spectra, respectively. All major bands of actin and myosin are represented in the actin-like and myosin-like spectra. The actin-like spectra appear to be less influenced by other cellular constituents than myosin. Thus, the cluster analysis can discriminate the actin and myosin regions in the tumor cell clusters during progression. Cluster analysis of actin and myosin-rich regions and corresponding spectra are reported in Supplementary Figs. 1 and 2. The distribution of F-actin in 2D PCa and 3D MSCs + PCa SC is shown in Supplementary Fig. 3. In a prior study we reported F-actin distribution in 2D PCa and 3D MSCs + PCa SC from the same testbeds as presented here^37^. The fluorescence imaging experiments in our study indicate actin distribution differences in individual cells in comparison with tumoroid cells at bone metastasis. The F-Actin is localized near cellular boundaries in the tumoroids compared to more even distribution of F-Actin in the as received single cells not at the bone metastisis site. Also, the concentration of F-actin per cell is reduced in prostate cancer bone metastasis samples in comparison to as received single cells.

Collectively, the observations from this study suggest that there are significant changes in the intensities of specific biomolecules relative to the total Raman-active constituents in 2D and 3D bone mimetic cultures. The 3D bone mimetic cultures mimic prostate cancer metastasis at the bone site^37,56^ We suggest the diagnostic potential of Raman microscopy for prostate cancer at the bone metastasis site. Prostate Imaging Reporting and Data System (PI-RADS) is used to inform the likelihood of a suspicious area being a clinically significant cancer. Based on future more extensive studies, we see the potential for using Raman imaging results to calculate PI-RADS scores or a similar quantitative reporting system for clinically relevant diagnosis.

## Conclusions

The present study marks the first application of Raman spectroscopy to classify 3D in vitro prostate cancer bone metastasis and to discriminate between cancer cells at different stages of tumorigenesis. Our results show that lipid, aromatic amino acid, and extracellular matrix components are involved in staging prostate cancer at the secondary bone site. Raman spectroscopic properties of prostate cancer metastasized at bone tissue can be effectively translated into a great wealth of diagnostic information providing new insights into biochemical and architectural changes of prostate cancer cells undergoing different stages of tumorigenesis. Also, from the point of clinical applications, it is possible to use hand-held Raman imaging of tissues ex vivo or in vivo for diagnostics at the patient level and would be very valuable for accessible cancers. The clinical application of Raman spectroscopy based endoscopy techniques for colon and prostate cancer diagnosis, and also the future application of the Raman signatures developed from this study for cancer staging and treatment response to therapy from biopsy samples address unmet needs towards new therapeutics for cancer. In addition,besides bone stabilizing drugs, clinicians use bone replacement materials and pins to stabilize damaged bone. It is of great interest to know the progression of metastasis in a patient to identify suitable therapies or combination thereof. Also from a scientific perspective, eventual in silico oncology approaches will need a dependable predictive capability of metastasis progression that is reliable for individual patients. The data collected with Raman imaging tools presented here in combination with nanomechanical evaluation of metastasis progression^36,37^ are valuable for development of predictive tools of cancer. Also, the use of Raman based tools enables a non destructive and in situ evaluation that is of great clinical importance. Our methods suggest that incorporating Raman spectroscopy and statistical techniques, such as PCA and cluster analysis, makes the classification of cancer progression straightforward.

## Methods

### Modification of MMT clay

The detailed procedure for the modification of Na-MMT clay is described elsewhere^57,58^. In brief, the 5-aminovaleric acid solution was added to preheated (60 °C) MMT suspension, and the mixture solution was kept for stirring. After one hour, the obtained slurry was centrifuged and washed to remove chloride ions, then dried at 70 °C, ground, and sieved to obtain a fine powder. Na-MMT clay was procured from Clay Minerals Respiratory at the University of Missouri, Columbia, and 5-aminovaleric acid was obtained from Sigma-Aldrich.

### Preparation of in situ HAPclay

We have followed the procedure described in our previous studies to prepare in situ HAPclay^27^. Briefly, the organically modified MMT clay powder was dissolved into Na_2_HPO_4_ solution (23.8 mM) by stirring at room temperature for two hours. Further, 39.8 mM of CaCl_2_ solution was added, and this suspension was stirred vigorously for eight hours (pH 7.4). The obtained precipitate was centrifuged, dried (70 °C), and subsequently ground and sieved to obtain a fine powder. Na_2_HPO_4_ and CaCl_2_ were purchased from JT Baker.

### Preparation of PCL/in situ HAPclay scaffolds

3D PCL/in situ HAPclay scaffolds were prepared according to the procedure described in earlier studies^24^. 3D PCL scaffolds were prepared with ten wt. % in situ HAP clay. In a typical procedure, the PCL solution was prepared by dissolving 3.6 g of polymer in 40 ml of 1,4-dioxane. Another solution was prepared by dissolving 0.4 g of prepared in situ HAPclay in 20 ml of 1,4-dioxane, followed by sonicating for 18 min. Freshly prepared in situ HAPclay solution was added to the polymer solution and stirred for two hours. We used the freeze extraction method to obtain 3D scaffolds. The chemicals PCL and 1, 4-dioxane were purchased from Sigma Aldrich.

### Cell culture

Human MSCs (PT-2501) (Lonza) were maintained in MSCGM™ Bulletkit™ medium (Lonza, PT3001). Human prostate cancer cell line MDA-PCa-2b (PCa) is purchased from ATCC and kept in 80% BRFF-HPC-1 (AthenaES) and 20% FBS (ATCC). For 2D cultures, cells were seeded on tissue culture-treated Petri dishes. For 3D sequential culture, MSCs were seeded at a density of 5 × 10^4^ cells per scaffold and cultured for 23 days to allow bone tissue formation. Further, prostate cancer cells PCa were seeded on newly formed bone tissue in the 3D bone mimetic scaffolds at a density of 5 × 10^4^ cells and maintained in 1:1 MSCs and PCa medium (Supplementary Fig. 4).

### Raman imaging

Raman spectra were acquired using a custom-built NIR confocal Raman microscopy system. The excitation source comprises a 785 nm wavelength from Ti: Sapphire laser (3900S, Spectra-Physics). The laser beam is focused on the sample, and backscattered light is collected using the same objective lens (1.2 NA, × 60, Olympus UPLASPO60XWIR). Raman signal is collected after a series of Rayleigh rejection filters by a collection fiber connected to a high-throughput imaging spectrograph (Holospec f/1.8i, Kaiser Optical Systems) with a thermoelectric-cooled, back-illuminated, and deep depleted CCD (PIXIS: 100BR_eXcelon, Princeton Instruments). The 785 nm wavelength is ideal for cell studies as it is just outside the visible range. Visible wavelengths cause degradation and photodamage to biological samples even at low power and short acquisition times. The primary goal of the present study is to investigate biochemical nuances at the cellular level, and thus a collection of cells in pellets from both 2D and 3D cultures was analyzed using point spectroscopic measurements. Briefly, after replacing the culture medium with PBS, cell pellets were formed by centrifugation and placed on top of the quartz coverslip followed by fixation with 4% paraformaldehyde for 10 min at room temperature, washing with phosphate-buffered saline (PBS) for 2–3 times before performing Raman experiments (Supplementary Fig. 4). Raman spectra were recorded for three locations from each sample. Spectra were collected from 76 µm × 76 µm areas in each location with about 2 µm step size. From each location, 1600 (40 × 40) spectra were acquired with 3 s integration time. We collected the data from a large size tissue that represents the average of data collected from several tumor cells to support the statistical significance and specificity of the signal. Wavelength calibration was performed before spectral acquisition by acquiring spectra from 4-acetamidophenol, a Raman scatterer with well-characterized peak positions. The 700–1800 cm^−1^ fingerprint region was used for the subsequent analysis (spectral resolution of 8 cm^−1^). Specially developed codes in MATLAB™ were used for simultaneous baseline correction of all Raman spectra in each image. The open-source Raman Tool Set was used for mapping the spectra. Subsequently, additional MATLAB™ codes were utilized for contour map development to allow uniform scaling across all maps generated for a given band. MATLAB™ codes were also developed to enable data to be transferred between Raman Tool Set and MATLAB™. Cosmic ray removal was also implemented before the spectra were used to create maps for predefined Raman bands in MATLAB (Mathworks Inc.). Further, 20 cell spectra from each sample were selected for intensity calculation of chosen Raman bands and principal component analysis. The mean ± standard deviation for the average spectrum obtained for each sample shown in Fig. 1A are presented in the Supplementary Fig. 5. All spectra were normalized to DNA band 780 cm^−1^ before analysis. Cancer cells, because of their rapid proliferation, possess multiple copies of DNA per cell in a given time compared to normal cells within the tumor. In addition, cancer cells are the predominant population compared to normal cells (endothelial cells, immune cells and fibroblasts, etc.,) in cancer tissues. Hence, we used Raman spectra respective to 780 cm^−1^ which represents the ring breathing of cytosine and thymine, the parts of DNA as internal control for normalization.

### Principal component analysis (PCA)

Principal Component Analysis (PCA) is an unsupervised statistical method that reduces variability by generating principal components (PCs). PCs are originated from a linear combination of original variables and are arranged based on the variance in the original dataset to explain correlation to one another. The first two PCs, i.e., PC1 and PC2, generally describe most of the data variance and generate a score plot. In a score plot, samples are positioned according to PC scores. For instance, samples with similar scores will take up a similar position while those with different scores will be placed in distance, thus making it easier to classify among samples^59^. PCA was conducted to determine whether spectra could be differentiated with respect to overall class 2D and 3D cultures. Before conducting PCA analysis, Raman spectra were imported and stored in a data matrix containing 867 rows (Raman shifts) and 80 columns (spectral intensities). PCA was performed in the spectral region of 700–1800 cm^−1^ using a covariance matrix (PCA for Spectroscopy, Origin Pro 2018).

### Cluster analysis

Cluster analysis is the method of grouping objects into clusters of similar items to distinguish and segregate a large volume of dissimilar data^60^. It is extensively used in data mining, machine learning, pattern recognition, image analysis, bioinformatics, and computer graphics in fields as diverse as astronomy, ecology, social sciences, and biology. Although there are many ways of conducting cluster analysis, we have combined cluster analysis with PCA to analyze spectral data from Raman mapping. Unsupervised algorithms such as principal component analysis and cluster analysis have been recently investigated for disease identification, including epithelial tissue tumors, brain tumors, skin tumors, bone diseases, atherosclerosis, kidney stones, gallstones, diabetes, and osteoarthritis^61^. A digital stripe of 5 μm width and 76 μm length is created outside of the Raman image for cluster analysis. The digital stripe comprises uniformly distributed pixels containing the reference spectra obtained separately for the pure constituents (actin or myosin). The reference spectra obtained from the Raman instrument are baseline corrected and scaled to the average cellular spectra using the 1444 cm^−1^ band for actin and 1002 cm^−1^ band for myosin. PCA was used to identify the different principal components. These principal components were then used to build clusters of the Raman data, followed by mapping. Specifically, we chose principle components PC1 to PC6 for actin and PC1 to PC9 for myosin. Next, the clusters were constructed using the CDP Rodriguez method^52^ in the Raman Toolset software. We used pure component spectra that were obtained from commercial sources of actin and myosin.

### Statistical analysis

All the data were expressed as mean ± standard deviation (n = 20), and the statistical significance (*p* value) was verified by one-way ANOVA followed by post hoc Tukey test for multiple comparisons (GraphPad Prism, v7.04).

## Supplementary Information

Supplementary Information.

## Data Availability

The datasets generated during and/or analysed during the current study are available from the corresponding author on reasonable request.
