# Supplementary material for: Label-free discrimination of tumorigenesis stages using in vitro prostate cancer bone metastasis model by Raman imaging
Source: Sci Rep. 2022 May 16;12:8050. doi: 10.1038/s41598-022-11800-w (PMC9110417; doi:10.1038/s41598-022-11800-w)
Supplement: Supplementary file 1 — Supplementary Information. [file 41598_2022_11800_MOESM1_ESM.docx]

Supplementary Information for

**Label-free discrimination of tumorigenesis stages using *in vitro* prostate cancer bone metastasis model by Raman imaging**

Sumanta Kar ^1^, Sharad V. Jaswandkar^1^, Kalpana S. Katti ^1^, Jeon Woong Kang ^2^, Peter T. C. So^2^, Ramasamy Paulmurugan^3^, Dorian Liepmann^4^, Renugopalakrishnan Venkatesan^5^, Dinesh R. Katti^1*^

*corresponding author. Email: [dinesh.katti@ndsu.edu](about:blank)


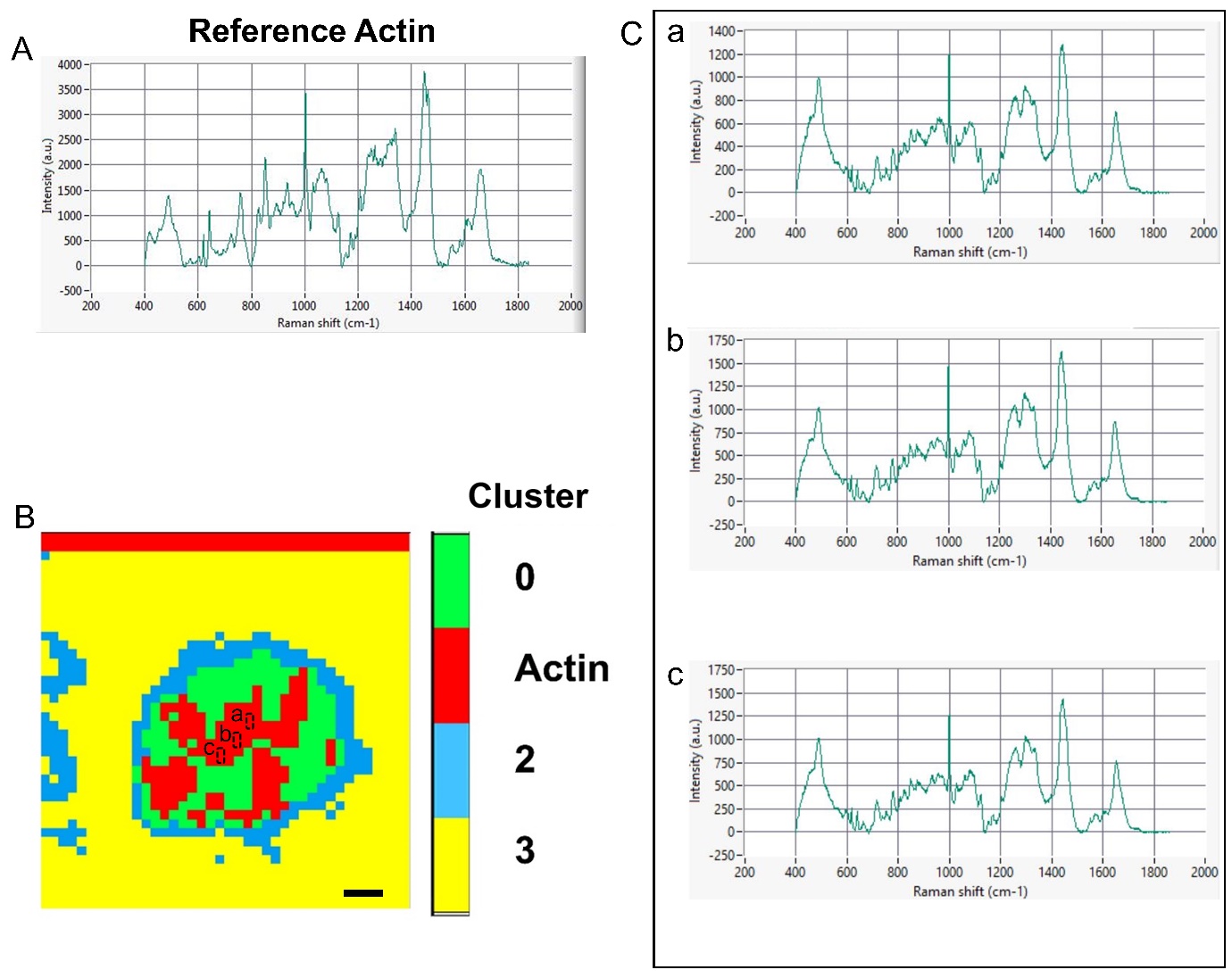


**Supplementary Figure 1.** Cluster analysis of Raman features of Actin. (A) Reference Actin spectra used for cluster analysis. (B) Cluster map of 3D d(23+15) MSCs+PCa SC indicating actin-rich regions colored in red and the red stripe on the top of the image constitutes digitally introduced reference actin, boxes (a,b, and c) drawn on the cluster map indicate a random location on the actin-rich region. Scale bar, 10 µm. (C) Spectra from a randomly chosen location on actin-region showing similar features as reference actin.


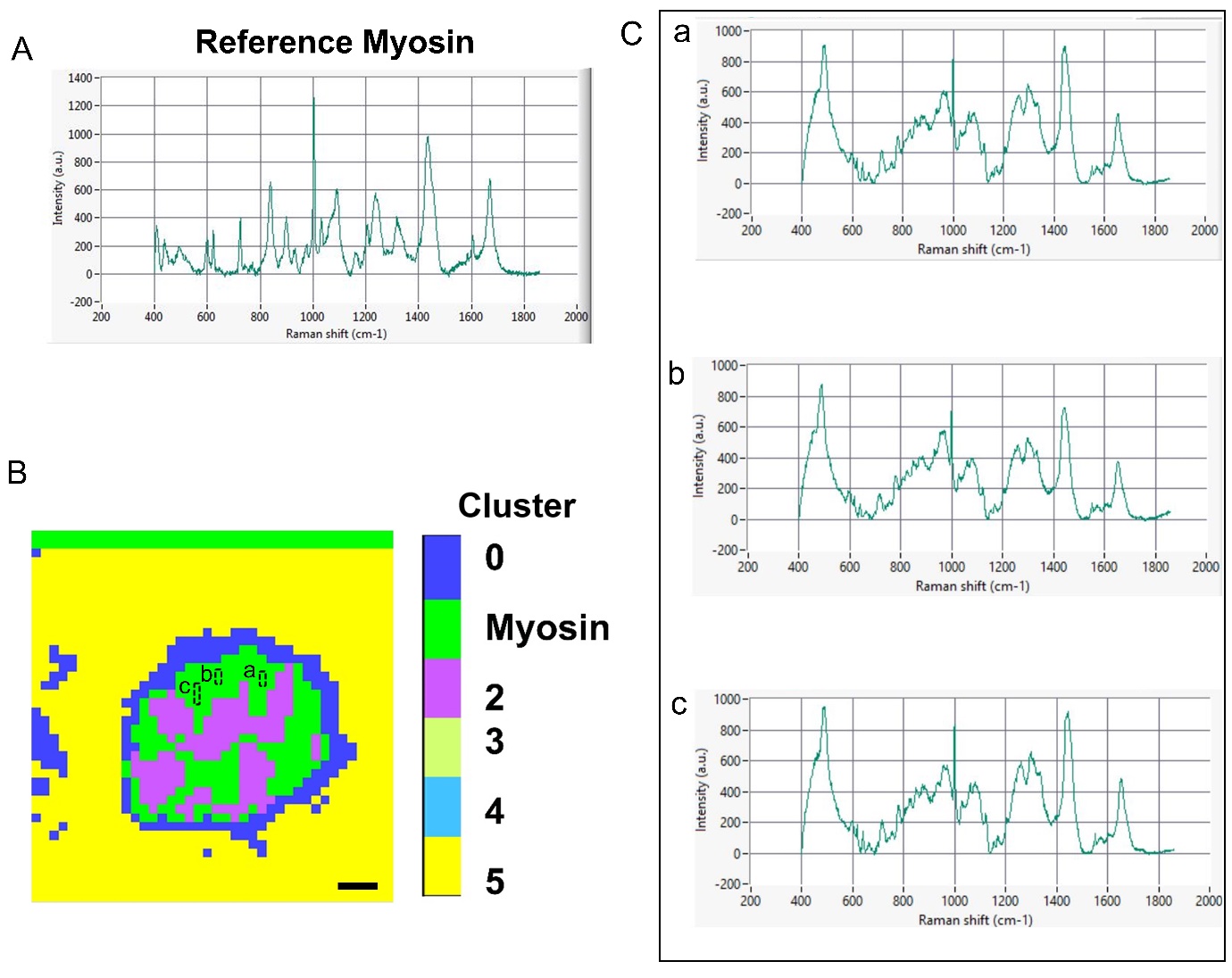


**Supplementary Figure 2.** Cluster analysis of Raman features of Myosin. (A) Reference Myosin spectra used for cluster analysis. (B) Cluster map of 3D d(23+15) MSCs+PCa SC indicating myosin-rich regions colored in green and the green stripe on the top of the image constitutes digitally introduced reference myosin, boxes (a,b, and c) drawn on the cluster map indicate a random location on the myosin-rich region. Scale bar, 10 µm. (C) Spectra from a randomly chosen location on myosin-region showing similar features as reference myosin.


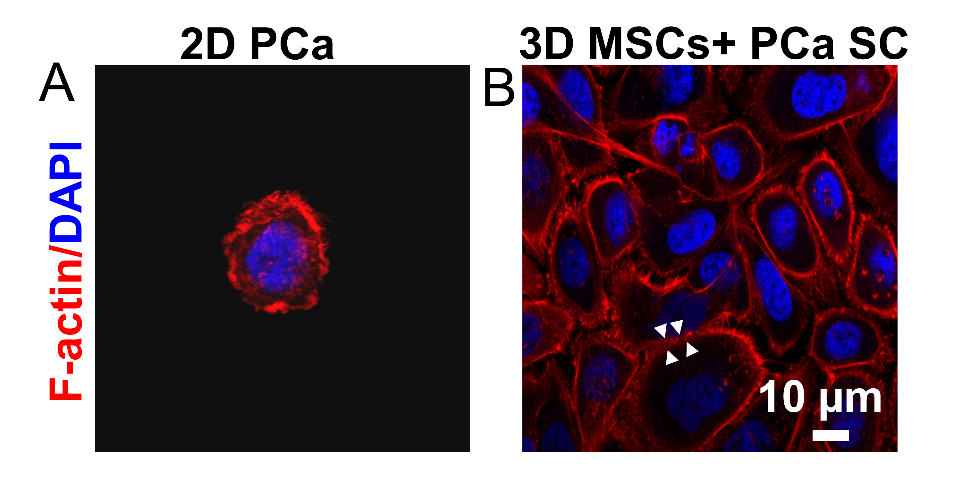


**Supplementary Figure 3.** Distribution of F-actin in prostate cancer cells. (A&B) Representative immunofluorescence images showing F-actin distribution in 2D PCa and 3D MSCs+PCa SC. Arrows indicate formation cells thin F-actin band (between adjacent cells. Briefly, cells were fixed with 4% paraformaldehyde in PBS, permeabilized with 0.2% TritonX-100 in PBS, and blocked with 0.2% fish skin gelatin (FSG). The actin cytoskeleton and nuclei of the cells were stained with Rhodamine Phalloidin and DAPI, respectively. The stained samples were washed with PBS and observed under a confocal microscope (Zeiss AxioObserver.Z1 LSM 700.). Scale bar, 10 µm.


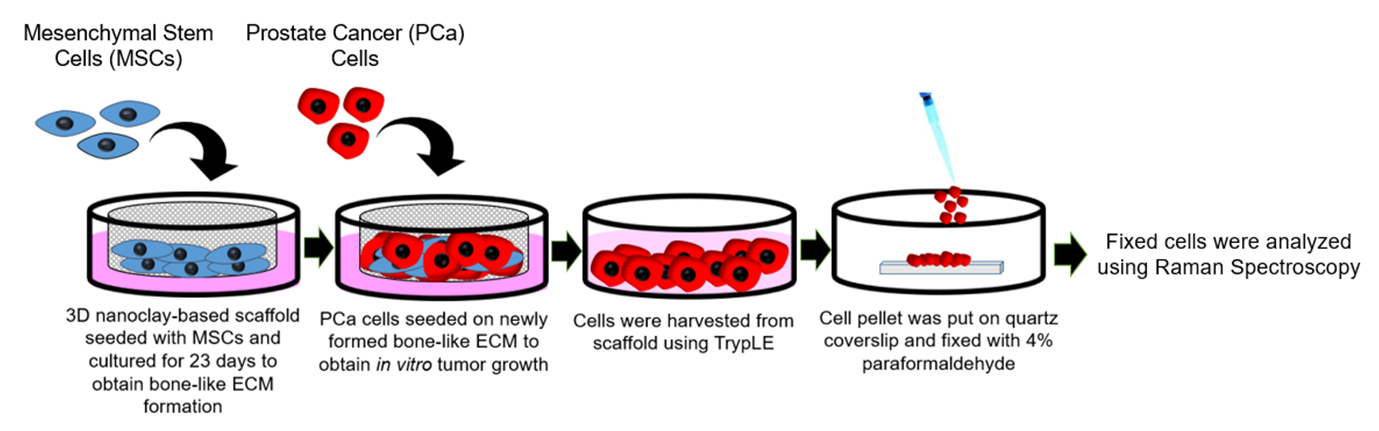


**Supplementary Figure 4.** Schematic showing the sequential culture of MSCs/prostate cancer cells and the workflow of Raman experiment. First, MSCs were grown on 3D nanoclay scaffolds for 23 days to allow bone tissue formation. Further, prostate cancer cells PCa were seeded on newly formed bone tissue in the 3D scaffolds to mimic prostate cancer bone metastasis. At the end predetermined culture period, cells were harvested from scaffolds and spun down to form a pellet. Then, the pellet was placed on top of the quartz coverslip followed by fixation with 4% paraformaldehyde and washing with PBS before performing Raman experiments


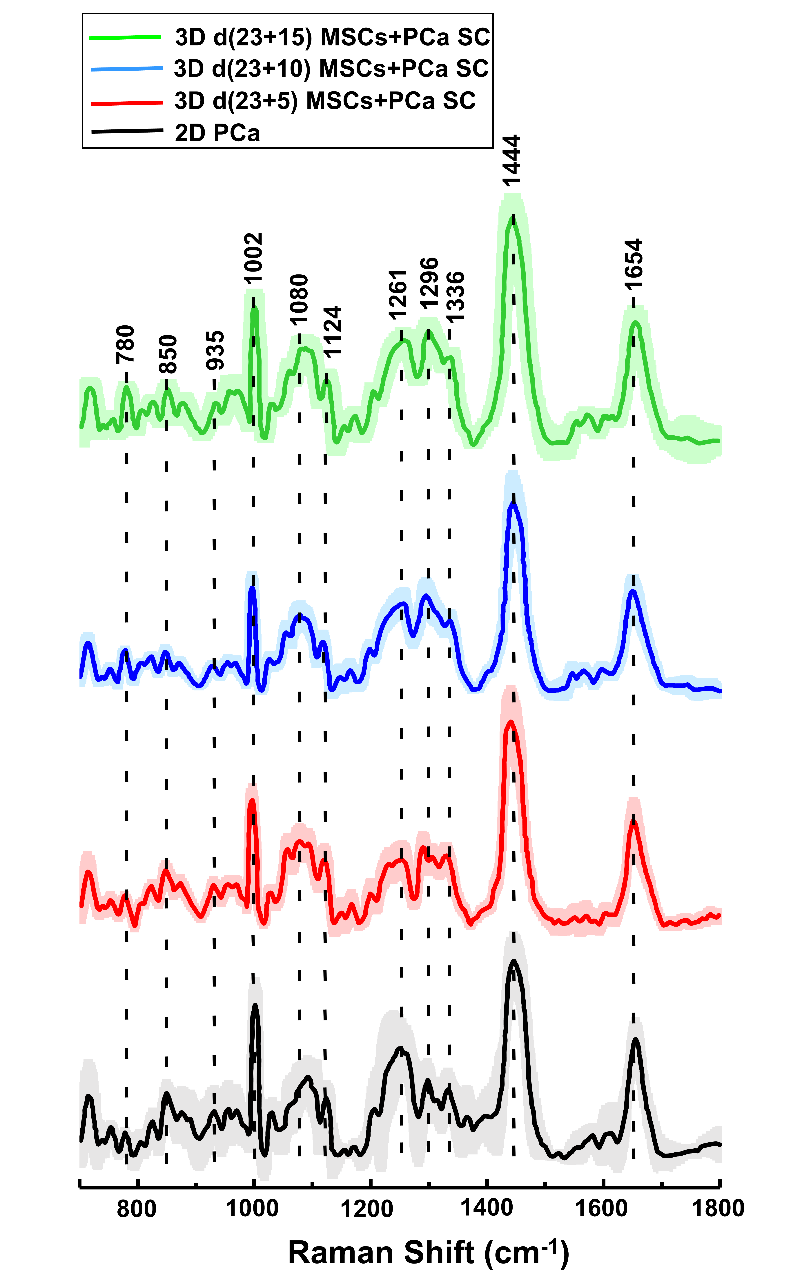


**Supplementary Figure 5.** Raman spectra of prostate cancer cells. Averages of 20 spectra from 4 samples (2D PCa and 3D MSCs + PCa SCs d(X+Y), where X=23 days of MSCs culture and Y= 5, 10, and 15 days of cancer cell culture after 23 days of MSCs culture) are shown in bold and overlaid on representative examples of spectra for each sample. Spectra are color-grouped according to culture type and duration of culture. The mean ± standard deviation for 20 spectra used for obtaining the average spectrum for each sample is superimposed in lighter shades of the color of the average spectra.
